# Oscillatory Photodynamic Therapy for Choroidal Neovascularization and Central Serous Retinopathy; a Pilot Study

**Published:** 2011-07

**Authors:** Gholam A Peyman, Michael Tsipursky, Nariman Nassiri, Mandi Conway

**Affiliations:** 1 Department of Ophthalmology, Tulane University, New Orleans, Louisiana, USA; 2 University of Arizona Biomedical Sciences, Phoenix, Arizona, USA; 3 Department of Ophthalmology, Northwestern University, Chicago, Illinois, USA

**Keywords:** Age-related Macular Degeneration, Central Serous Retinopathy, Choroidal Neovascularization, Oscillatory Photodynamic Therapy, Verteporfin

## Abstract

**Purpose:**

To report the preliminary results of oscillatory photodynamic therapy (OPDT) for choroidal neovascularization (CNV) and central serous retinopathy (CSR).

**Methods:**

This study included 7 eyes of 6 patients with CSR (2 eyes), idiopathic CNV (2 eyes), CNV due to age-related macular degeneration (AMD) (2 eyes), and peripapillary CNV secondary to presumed ocular histoplasmosis syndrome (1 eye). Intravenous verteporfin (6 mg/m^2^ body surface area) was infused over 10 minutes followed by oscillating laser (wavelength 689 nm) covering slightly beyond the entire lesion. An Area Centralis lens was applied and laser was delivered (600 mW/cm^2^ fluence rate and 50 J/cm^2^ dose). Intravitreal bevacizumab and dexamethasone combination therapy was used with OPDT in 4 eyes with CNV; intravitreal dexamethasone and triamcinolone acetonide were injected in the other eye with CNV. Clinical examination, funduscopy, fluorescein angiography, and optical coherence tomography (OCT) were performed at baseline and after treatment.

**Results:**

After mean follow-up of 7.1±5.1 months, visual acuity improved from 0.87±0.69 logMAR (20/160) to 0.60±0.65 logMAR (20/80) (P = 0.027); central foveal thickness decreased from 322±62.1 to 240.7±34.8 microns as measured by OCT (P = 0.018). Fluorescein angiography and OCT demonstrated cessation of vascular leakage, and resolution of hemorrhage and subretinal fluid in all eyes. No adverse events or recurrence were noted.

**Conclusion:**

OPDT was effective in treating CNV lesions and CSR. OPDT may be an improvement on standard PDT due to reduced side effects, thermal damage and scarring.

## INTRODUCTION

Photodynamic therapy (PDT) has been used successfully to treat choroidal neovascularization (CNV) secondary to high myopia, age-related macular degeneration (AMD), and ocular histoplasmosis syndrome (OHS).^1^–^3^ It entails the intravenous administration of verteporfin (Visudyne; Novartis AG, Basel, Switzerland). CNV membranes can be treated with a red diode laser (689 nm wavelength), administered to the CNV, locally activating the verteporfin in the area covered by the laser. The use of a nonthermal laser in photodynamic therapy (PDT) may avoid the risk of permanent destruction of the adjacent neurosensory retina as seen with conventional photocoagulation therapy.

The retinal pigment epithelium (RPE) and choriocapillaris, which together constitute the blood-retinal barrier (BRB),^4^ play a pivotal role in the viability and functionality of the neurosensory retina.^5^ RPE changes may adversely affect photoreceptor function and survival due to disruption of the BRB and leakage of fluid into the subretinal space. PDT has been shown to induce structural changes in the RPE both experimentally^6^–^10^ and clinically^11^–^13^. Observed changes in the RPE and choriocapillaris depend on light intensity, duration of exposure, concentration of the photosensitizer, and interval between dye administration and laser therapy.^8^

Mennel et al^7^ reported that the combination of a therapeutic concentration of verteporfin and application of non-thermal laser led to a morphologically and functionally detectable breakdown of the outer BRB function of the RPE without damage to RPE cells in vitro. However, they stated that increasing the concentration of verteporfin (2 mg/ml) resulted in RPE cell damage. Several factors were reported to influence verteporfin concentration adjacent to RPE cells including blood flow, low density lipoprotein (LDL) uptake, concentration of LDL receptors, size, location and type of CNV, and leakage. Persistent RPE cell destruction is more severe in younger subjects,^11^,^12^ which can be due to better perfusion (higher dosage of verteporfin at the RPE) as well as clearer media (greater activation of verteporfin by the laser)^14^. Animal models have revealed other factors which may influence the effectiveness of PDT including media opacity,^14^ intraocular pressure, location of treatment within the fundus, equivalent fluence (lower energy and longer duration), and retreatment as well as fundus pigmentation.^15^

The non-thermal laser used in PDT, like thermal lasers, can induce alterations in the RPE and breakdown of the BRB, resulting in dysfunction of the neurosensory retina. Such PDT-induced RPE damage might be reduced by individualized treatment that takes into account parameters such as media transparency, age and gender, and optimized laser energy dosage. For example, reduced-fluence PDT has been reported to be effective^16^–^18^ in terms of visual outcomes and safer than standard PDT regarding choroidal alterations^17^,^19^ as well as RPE changes.^18^ Sacu et al^19^ reported that reduced-fluence PDT is more effective than standard photodynamic therapy. Additionally, Azab et al^20^ reported a 3-line loss of visual acuity in 14% of eyes assigned to reduced-fluence PDT as compared to 28% of eyes undergoing standard PDT.

PDT is now infrequently used as monotherapy for AMD; it is most often used in combination with other treatment modalities.^21^–^25^ Reduced laser dose and verteporfin concentration may be achieved by the simultaneous use of intravitreal triamcinolone^26^,^27^ or anti-vascular endothelial growth factor (VEGF) agents. The latter counteract the effect of VEGF,^28^,^29^ which is known to be increased in PDT-treated area.^30^,^31^ On the other hand, the addition of reduced laser dose PDT (12 or 25 J/cm^2^) to bevacizumab therapy has been shown to decrease the number of bevacizumab treatments.^32^

Peyman et al^33^ used indocyanine green (ICG) assisted oscillatory thermotherapy (OTT) at individualized subthreshold energy levels to elicit primarily a photodynamic effect from the laser while reducing the thermal effect. This was achieved by applying the predetermined subthreshold thermal energy level in an oscillatory mode instead of the standard stationary mode. OTT prevents accumulation of thermal energy in the tissues, permitting choriocapillary blood flow and convection to cool down heated tissues, thus avoiding potential photocoagulative damage. This approach allows treating the retina for an extended period of time, thereby providing more ICG-induced photodynamic effect.

In this pilot study, we studied the primary outcomes of oscillatory photodynamic therapy (OPDT) using verteporfin. We believe that the oscillatory mode allows more precise and customized treatment of the lesion. It provides flexibility in treating areas of pathology without extending treatment into unaffected tissues; at the same time it allows prolonged treatment over the neovascular membrane.

## METHODS

Consent for off-label treatment was obtained after consultation with the Ophthalmic Medical Insurance Company (OMIC). This prospective study was approved by the Tulane University Institutional Review Board. Seven eyes of 6 female patients underwent OPDT between September 2008 and December 2009. Underlying abnormalities included central serous retinopathy (CSR, 2 eyes), idiopathic CNV (2 eyes), CNV due to AMD (2 eyes), and peripapillary CNV from presumed OHS (1 eye). Two eyes (cases #3 and 6) had history of treatment with anti-VEGF agents and one eye (case #4) had frequent recurrences, initially treated with thermal laser as well as anti-VEGF therapy. Complete ophthalmologic examination at baseline, 2 weeks post-treatment, and monthly thereafter included assessment of visual acuity, fundus examination (non-contact 90-diopter lens), color fundus photographs, and optical coherence tomography (OCT). Fluorescein angiography (IMAGEnet System, Topcon, Tokyo, Japan) was performed at baseline and repeated based on clinical findings. Visual acuity was measured with a Snellen chart (CP-690, Nidek, Gamagori, Japan) calibrated for 20 feet (6 meters) by the line assignment method and converted to logMAR notations by the Standard Conversion Table for statistical analyses.

Intravenous verteporfin (6 mg/m^2^ body surface area) was infused over 10-minutes. The Zeiss Visulas 690s laser system (689-nm wavelength, Carl Zeiss Meditec Inc., Dublin, California, USA) was used to treat the lesion for 83 seconds (except case #5 who received 166 seconds of treatment). In all cases, an Area Centralis lens was applied and laser was delivered at a standard fluence of 600 mW/cm^2^ and dose of 50 mJ/cm^2^. During the procedure, the operator kept the fundus contact lens steady while oscillating the laser beam at 2–3 Hertz using a spot size equal to half the size of the lesion to cover the entire lesion. Precautions for exposure to light were reviewed with the patient, who was instructed to stay out of sunlight and excessive light exposure for 5 days. The treatment was well tolerated and no complications were observed.

Intravitreal injections of bevacizumab (1.25 mg) and dexamethasone (1 mg) were performed at the same session in 4 eyes with CNV. In one eye with recalcitrant idiopathic CNV and a possible episode of post-anti-VEGF stroke (case #4), intravitreal dexamethasone (360 mcg) and triamcinolone acetate (400 mcg) were injected.

## RESULTS

Baseline variables are summarized in Table 1. Mean follow-up was 7.1 ± 5.1 months. Mean visual acuity improved from 0.87 ± 0.69 logMAR (20/160) to 0.58 ± 0.65 logMAR (20/80) after the procedure (Wilcoxon signed-rank test, P = 0.027, Table 1). Central subfoveal thickness on OCT decreased from 322.3 ± 62.1 μm at baseline to 240.1 ± 34.8 μm after the procedure (Wilcoxon signed-rank test, P = 0.018). Volumetric measurements in case #5 showed that pigment epithelial detachment/scar and subretinal fluid were significantly reduced.

There were no instances of infusion-related back pain, photosensitivity reactions, or injection site adverse events. Two representative cases are described below in detail.

### Case 1

**Figure 1:**
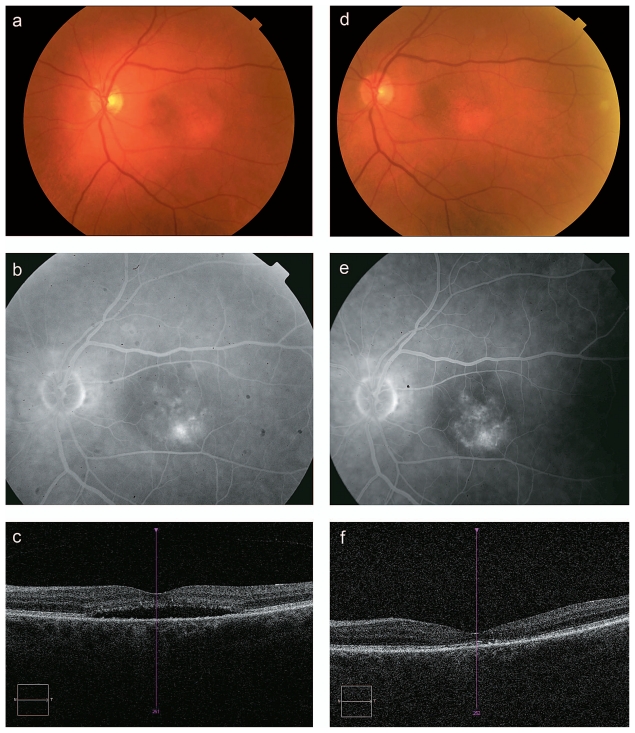
A 70-year-old female patient with chronic central serous retinopathy in the left eye (case #1). Pre-treatment: Color fundus photograph shows serous neurosensory detachment of the macula with pigment mottling (a). Fluorescein angiography shows multifocal areas of increasing hyperfluorescence in the inferior temporal macula consistent with leakage (b). OCT confirms subretinal fluid (central subfoveal thickness 310 μm) with loss of photoreceptor cells (c). Post-treatment: Color fundus photograph (d), fluorescein angiography (e) and OCT (central subfoveal thickness 180 μm) (f) all demonstrate resolution of the serous detachment with a similar amount of pigmentary changes in the macula.

The first patient was a 70-year-old lady with longstanding CSR in her left eye and no history of treatment. Pigment mottling was evident on color fundus photography with angiographic activity in the inferotemporal macula. Subretinal fluid was present on OCT with serous neurosensory detachment in the macula (Figures 1a–c). At baseline, visual acuity was 20/100 and OCT demonstrated central subfoveal thickness of 310 μm with loss of photoreceptor inner and outer segments. She was treated with OPDT and verteporfin (2400-μm spot size, 600 mW/cm^2^ fluence rate, and 50 mJ/cm^2^ dose for 83 seconds). After treatment, the serous detachment resolved and the patient remained clinically stable as determined by angiography and OCT (Figures 1d–e). Visual acuity improved to 20/40 without metamorphopsia and central subfoveal thickness was decreased to 180 μm. Due to disruption of the photoreceptor layer and RPE attenuation due to longstanding neurosensory detachment, recovery of visual acuity was incomplete. The patient remained stable up to 11 months.

### Case 3

**Figure 2:**
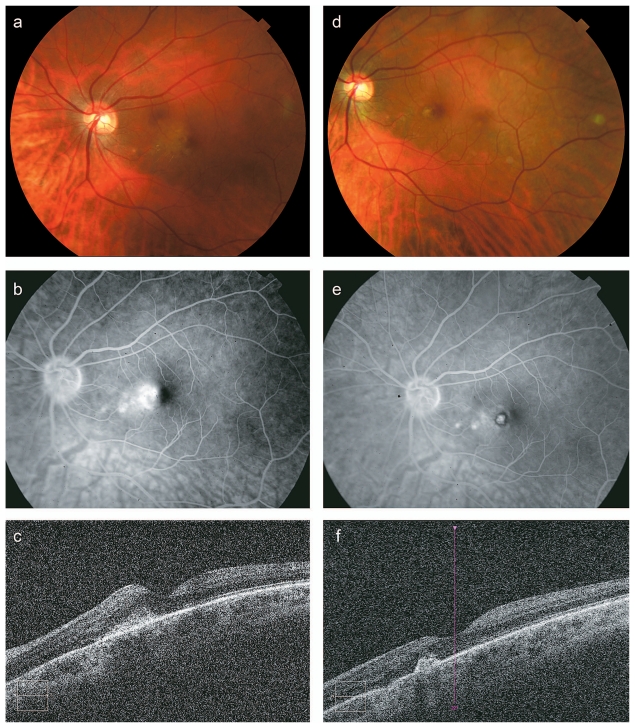
A 34-year-old female patient with idiopathic choroidal neovascular membrane (CNVM) in the left eye (case #3). Pre-treatment: Color fundus photograph shows deep subretinal hemorrhage associated with retinal thickening and lipid exudation in the nasal macula (a). Fluorescein angiography demonstrates multiple leakage sites from the CNVM in the nasal macula (b). OCT confirms retinal thickening in the nasal fovea with a hyper-reflective subretinal lesion (central subfoveal thickness 263 μm) (c). Post-treatment: Color fundus photograph (d), fluorescein angiography (e), and OCT (central subfoveal thickness 250 μm) (f) all show a consolidated subretinal scar without persistent leakage or associated retinal thickening, consistent with involution of CNVM. OCT from the adjacent area shows slightly disrupted but mostly preserved photoreceptor layer despite OPDT. The scar appears hyperfluorescent and there is a ring of hypofluorescence corresponding to blockage from pigment migration. There are two small areas of hyperfluorescence nasal to the fovea consistent with window defects.

This 34-year-old lady had mild myopia and reduced visual acuity in her left eye. She presented with a grayish subretinal lesion in the nasal fovea associated with subretinal hemorrhage, exudation and retinal thickening extending into the center of the fovea. Leakage and retinal thickening in the nasal fovea were confirmed with fluorescein angiography and OCT (Figures 2a–c). She was diagnosed with idiopathic choroidal neovascular membrane, which did not respond to an injection of intravitreal bevacizumab. At baseline, visual acuity was 20/40 and central subfoveal thickness was 263 μm. She was treated with OPDT and verteporfin (800-μm spot size for 83 seconds) as well as adjunctive intravitreal bevacizumab/dexamethasone. After treatment the patient had a consolidated subretinal scar in the nasal fovea without persistent leakage on angiography, or fluid on OCT which demonstrated central subfoveal thickness of 250 μm (Figures 2d–f). The treated perifoveal retina showed relatively preserved photoreceptor structure on OCT. Visual acuity improved to 20/25 without metamorphopsia. There was no recurrence up to 5 months after treatment.

## DISCUSSION

Herein, we report the preliminary outcomes of OPDT with a strong photosensitizer, verteporfin in 7 eyes with CNV or CSR. This report describes a novel application of PDT in an oscillatory fashion. The current realistic goal of PDT is to retard progression of CNV due to AMD and other causes, and possibly restore normal vision without causing significant scarring. We believe that oscillatory PDT reduces the risk of retinal pigment epithelial damage since it decreases total fluence which itself depends on the speed of the oscillation. By using small spot size and moving it over the treatment area, one can avoid treatment of healthy retina; this advantage is especially marked for irregularly shaped lesions.

The primary outcome measure in PDT studies is to assess the proportion of eyes that avoid moderate visual loss (loss of fewer than 3 lines or 15 letters).^1^ Our clinical outcomes with mean follow-up of 7 months showed that OPDT with verteporfin was successful in improving central vision in all eyes except one (case #7), in which the 1-line reduction in visual acuity could be attributed to progression of the disease or increased cataract. The remaining cases showed 37.4% improvement in visual acuity equivalent to 3 Snellen lines. Additionally, post-treatment findings on funduscopy, fluorescein angiography, and OCT were suggestive of cessation of vascular leakage as well as resolution of hemorrhage and subretinal fluid in all cases.

Cardinal features of PDT include the coexistence of a sensitizer, light, and oxygen. The main mechanism of action of PDT is vascular occlusion due to damage to endothelial cells and subsequent thrombosis of both neovascular and normal choriocapillaris.^34^,^35^ The response to PDT appears to be caused by a combination of direct cytotoxicity to vascular endothelial cells, subsequent platelet adhesion and degranulation, thrombosis, and vasoconstriction, leading to blood flow stasis and vaso-occlusion of the choriocapillaris. PDT exerts its cytotoxic effect by generation of reactive oxygen species, which can induce cell death either by apoptosis or necrosis; it can even initiate a remodeling response.^34^,^35^ This vascular reaction has been associated with variable damage to the RPE and photoreceptors.^6^–^13^

Application of the laser in an oscillatory fashion can potentially reduce damage to the RPE by reducing overall fluence. A laser beam with spot size of approximately one half the size of the lesion is moved 2–3 times per second in continuous fashion over the entire treatment area. The term fluence takes into account the energy level used for treatment, with laser spot and application time for coverage of the entire lesion in stationary fashion. It can be estimated that if the laser is in any particular spot for about 0.5 second during the 2 Hz oscillatory mode, total fluence is approximately reduced to half the standard method of application. The power setting in our series (600 mW/cm^2^) and application time (83 seconds) were equal to a standard protocol. No visible whitening or subsequent fibrotic reaction was observed from OPDT application. We used bevacizumab and dexamethasone in combination with OPDT for most of our patients.^36^ Dexamethasone, and in one case, triamcinolone acetate, was added to control the inflammatory response to laser therapy.^29^

Peyman et al^15^ showed that PDT retreatment resulted in progressive thinning of the neurosensory retina with loss of photoreceptor outer segments and nuclei in the rabbit eye. In the current study, no patient required retreatment and no significant loss of photoreceptors was observed on OCT. This outcome can be due to both the oscillatory mode of PDT application and triple therapy. Triple therapy significantly reduces the number of treatments.^22^,^37^–^39^ It is noteworthy that three treated eyes had been recalcitrant to previous anti-VEGF therapy (Table 1).

OPDT is an improved mode of applying standard treatment allowing greater activation of the photosensitizer and less cytotoxic damage to the neuroretina due to reduced fluence. This is evidenced by the lack of a “burn spot” or loss of photoreceptors on post-treatment color images, fluorescein angiography, and OCT. It is impossible to completely prevent the recurrence of CNV in choroidal disease, especially in AMD. Thus, there will likely be subjects who require retreatments. Standard PDT retreatments can cause scarring and fibrosis,^40^,^41^ but we expect this to be less likely with oscillatory PDT.

PDT with verteporfin has been effective for chronic CSR by improving visual acuity and reducing subretinal fluid.^42^–^47^ PDT treatment for CSR causes short-term choriocapillaris hypoperfusion and long-term choroidal vascular remodeling, leading to reduction in choroidal congestion, vascular hyperpermeability, and extravascular leakage.^42^,^48^,^49^ However, complications such as secondary CNV, persistent choriocapillaris hypoperfusion, and pigmentary RPE changes in the treated zone have been reported.^42^,^46^,^50^,^51^ Modified PDT protocols in terms of verteporfin dosage, fluence rate, time course of delivery, or a combination thereof have been suggested.^52^ Reibaldi et al^53^ showed that low-fluence PDT is effective in long-standing chronic CSR with foveal and gravitational atrophy of the retina and reported functional improvement without significant retinal or choroidal damage. In a comparative study^17^ they reported that both standard- and low-fluence PDT resulted in complete subretinal fluid reabsorption and visual improvement. PDT-related choroidal hypoperfusion could be reduced by low-fluence PDT.

OPDT offers the choice of early treatment for CSR; this may prevent atrophy of photoreceptors caused by long-standing subretinal fluid leading to compromised retinal function. Two subjects in this study had chronic CSR (cases #1 and #2). They both showed significant improvement in visual acuity as well as resolution of subretinal fluid with a single OPDT treatment, without PDT-related side effects and need for retreatment. The chronic nature of subretinal fluid was the reason for incomplete recovery of visual acuity. There was no significant disruption of the neuroretina on post-treatment OCT.

This pilot study reports on the outcomes of therapy with a novel method of oscillatory PDT with verteporfin. Standard PDT has fallen out of favor due to the success of anti-VEGF therapy. However, drawbacks to repeated intraocular injections include the risk of endophthalmitis and retinal detachment, as well as an overwhelming cost to healthcare systems. OPDT may be applied for all CNV lesions and reduce the need for repeat injections. Even though there are reports of CSR responding to anti-VEGF therapy, there are recalcitrant cases that will still require laser treatment.^54^,^55^ OPDT appears to be an improved method of administering PDT and is effective in treating CNV lesions and CSR. It may be superior to standard PDT because of reduced total fluence and enhanced photodynamic effect. Furthermore, it allows the operator to customize treatment over the lesion, potentially spending more time over more aggressive components of the choroidal neovascular membrane. Smaller spot size also reduces inadvertent treatment of normal retina which may occur with irregular shaped lesions.

## Figures and Tables

**Table 1:** Pre- and post-treatment data for six female patients (7 eyes) undergoing oscillatory photodynamic therapy (OPDT) with verteporfin

No.	Age (yr)	History of Treatment	R/L	Diagnosis	F/u (mo)	VA (LogMAR)		Spot Size (μm)	OCT measurement (μm)		Adjunctive treatments	Duration (s)	Details
						Pre	Post		Pre	Post			
1	70		L	CSR	11	20/100 (0.7)	20/40 (0.3)	2400	310	180	none	83	Hypercholesterolemia, ocular hypertension (on Alphagan)
2	62		L	CSR	3.5	20/50 (0.4)	20/30 (0.2)	3000	343	207	Intravitreal Avastin (1.25 mg)	83	
3	34	Avastin (x1)	L	Idiopathic CNV	5	20/40 (0.3)	20/25 (0.1)	800	263	250	Intravitreal Avastin (1.25 mg)/Decadron (1 mg)	83	
4	52	Argon; Avastin (x3); Lucentis (x2); Conventional PDT	R	Idiopathic recurrent CNV	17	20/200 (1)	20/40 (0.3)	2000	450	253	Intravitreal Decadron (360 mcg)/Kenalog (400 mcg)	83	HTN; hypothyroid; possible post-anti-VEGF stroke
5	70		L	CNV from AMD	6	20/200 (1)	20/100 (0.7)	800	305 *2.31 mm^3^	272 *0.78 mm^3^	Intravitreal Avastin (1.25 mg)/Decadron (500 mcg)	83	HTN; hyperlipidemia; heavy smoker; coronary artery disease
6	71	Avastin (x3)	R	Macular CNV from AMD	3	1/200 (2.3)	2/200 (2)	1200	311	276	Intravitreal Avastin (1.25 mg)/Decadron (1 mg)	83	HTN; hypothyroid; glaucoma (Travatan)
7	71		L	Peripapillary CNV from POHS	4	20/50 (0.4)	20/60 (0.5)	900	274	243	Intravitreal Avastin (1.25 mg)/Decadron (1 mg)	83	5 years ago gamma knife for trigeminal neuralgia on the left side

yr, year; R, right; L, left; F/U, follow-up; mo, month; VA, visual acuity; Pre, pre-treatment; Post, post-treatment; OCT, optical coherence tomography; CSR, central serous retinopathy; CNV, choroidal neovascularization; PDT, photodynamic therapy; HTN: hypertension; AMD, age-related macular degeneration; POHS, presumed ocular histoplasmosis syndrome.

* Volumetric measurement shows significant reduction in overall volume (including pigment epithelial detachment and subretinal fluid) after treatment.
